# Phase I Clinical Study with the GRPR-Antagonist [^99m^Tc]Tc-DB8 for SPECT Imaging of Prostate Cancer: Does the Injected Peptide Mass Make a Difference?

**DOI:** 10.3390/pharmaceutics17101323

**Published:** 2025-10-12

**Authors:** Anna Orlova, Anastasia Rybina, Anna Medvedeva, Roman Zelchan, Olga Bragina, Liubov Tashireva, Maria Larkina, Ruslan Varvashenya, Nadejda Lushnikova, Panagiotis Kanellopoulos, Theodosia Maina, Berthold A. Nock, Vladimir Tolmachev, Vladimir Chernov

**Affiliations:** 1 Department of Medicinal Chemistry, Uppsala University, 751 23 Uppsala, Sweden; panagiotis.kanellopoulos@ilk.uu.se; 2 Science for Life Laboratory, Uppsala University, 752 37 Uppsala, Sweden; 3 Department of Nuclear Medicine, Cancer Research Institute, Tomsk National Research Medical Center, Russian Academy of Sciences, 634009 Tomsk, Russia; pankovaan@mail.ru (A.R.); medvedeva@tnimc.ru (A.M.); r.zelchan@yandex.ru (R.Z.); rungis@mail.ru (O.B.); chernov@tnimc.ru (V.C.); 4 Research Centrum for Oncotheranostics, Research School of Chemistry and Applied Biomedical Sciences, Tomsk Polytechnic University, 634050 Tomsk, Russia; marialarkina@mail.ru; 5 Department of General and Molecular Pathology, Cancer Research Institute, Tomsk National Research Medical Center, Russian Academy of Sciences, 634009 Tomsk, Russia; lkleptsova@mail.ru; 6 The Laboratory of Molecular Therapy of Cancer, Cancer Research Institute, Tomsk National Research Medical Center, Russian Academy of Sciences, 634028 Tomsk, Russia; 7 Department of Pharmaceutical Analysis, Siberian State Medical University, 634050 Tomsk, Russia; 8 Department of General Oncology, Cancer Research Institute, Tomsk National Research Medical Center, Russian Academy of Sciences, 634009 Tomsk, Russia; 9 Molecular Radiopharmacy, INRaSTES, NCSR “Demokritos”, 15341 Athens, Greece; maina_thea@hotmail.com (T.M.); nock_berthold.a@hotmail.com (B.A.N.); 10 Department of Immunology, Genetics and Pathology, Uppsala University, 752 37 Uppsala, Sweden; vladimir.tolmachev@igp.uu.se

**Keywords:** GRPR, SPECT, prostate cancer, Tc-99m, molecular imaging

## Abstract

**Background/Objectives**: The gastrin-releasing peptide receptor (GRPR) shows high-density expression in prostate cancer (PCa), especially in the early stages of the disease. The introduction of a safe radiotracer for assessing GRPR-expression in PCa may serve as an alternative or complementary tracer to PSMA-directed probes for patients with insufficient PSMA expression. In the present study, the tolerability and safety, biodistribution, and dosimetry of the new GRPR-targeting radiopeptide [^99m^Tc]Tc-DB8 were investigated for the first time in male PCa patients. A mass escalation study was performed, aiming to improve tumor-to-background contrast and, thereby, to enhance diagnostic accuracy. **Methods**: Sixteen male patients were enrolled in a single-center diagnostic open-label exploratory Phase I clinical trial. Patients were administered a single intravenous injection of 40, 80, or 120 µg of [^99m^Tc]Tc-DB8 peptide (n = 5–6) and underwent whole-body planar imaging (anterior and posterior) 2, 4, 6, and 24 h post-injection (pi) and SPECT-CT acquisition 2, 4, and 6 h pi. **Results**: Administration of [^99m^Tc]Tc-DB8 was well tolerated at all tested peptide masses. The effective dose did not differ significantly between the injected peptide mass and was 0.005 ± 0.003 mSv/MBq. High activity uptake was observed in the pancreas and kidneys, which 3-fold decreased with an increasing injected peptide mass from 40 to 120 µg. The activity uptake in primary tumors did not differ significantly between cohorts injected with different peptide masses [SUV_max_ 1.65–9.96]. The tumor-to-muscle ratios increased with time and were the highest for the cohort injected with 120 µg of peptide, 7.2 ± 3.1 (4.64-11-25) at 4 h pi. **Conclusions**: Single intravenous administration of [^99m^Tc]Tc-DB8, for visualization of GRPR expression in PCa using SPECT imaging was well tolerated in a peptide mass range of 40–120 µg. An injected peptide mass of 80–120 µg/patient and SPECT acquisition 2–4 h pi were found to be optimal for further clinical studies due to the significantly lower activity accumulation in the pancreas and kidneys.

## 1. Introduction

Almost one quarter of diagnosed cancers in men in Europe are prostate cancers (PCa). PCa is the third most common cancer-specific cause of death in males in this region (10%) [1]. Understandably, staging and choice of appropriate treatment are keys to better therapeutic management and outcomes. Measurements of biomarkers in the blood, especially of prostate-specific antigen (PSA), have been instrumental in PCa diagnosis combined with other diagnostic tools, such as imaging and biopsy. In recent years, restaging of human PCa after initial therapy has been increasingly relying on radiolabeled choline and positron emission tomography (PET) [2], whereas disease metastasized in the bone can be visualized with bone-seeking agents [3].

Nuclear medicine has revolutionized both the diagnosis and treatment of cancer with the advent of molecular probes directed to specific cancer-associated targets, dynamically entering the PCa management [4]. The molecular probes are designed for single-photon emission computed tomography (SPECT), labeled with photon-emitting nuclides (mainly Tc-99m), or for positron emission tomography (PET), labeled with positron emitters (e.g., F-18, Ga-68), enabling the non-invasive diagnosis and staging of the disease. Along these lines, prostate-specific membrane antigen (PSMA) inhibitors labeled with a variety of radionuclides have gained momentum in PCa diagnosis and therapy due to the high-density expression of PSMA in PCa [5,6]. This allows for patient stratification for specific therapies, as well as selection of patients eligible for targeted radiotherapy with α- or β-particle emitters (Lu-177, Ac-225) to deliver radiotoxic payloads to PCa lesions expressing the target. A great number of such radioligands have been emerging in recent days, with some probes already approved by authorities and widely used in the clinic [7]. The recent wide clinical use of anti-PSMA radioligands has inadvertently revealed a number of shortcomings, including unfavorable uptake in certain normal organs (e.g., the kidneys the salivary and lacrimal glands) or false-positive uptake in non-cancerous foci in the thorax. Furthermore, PSMA-expression levels tend to be low in primary lesions and subgroups of advanced PCa lesions, compromising diagnostic accuracy [8,9,10,11].

Concurrently, alternative molecular targets have been continuously investigated to be used in the diagnosis and therapy of PCa. Of particular interest is the gastrin-releasing peptide receptor (GRPR), which has been attracting considerable attention in the nuclear medicine community, owing to its high-density expression in a multitude of frequently occurring malignant tumors [12,13]. The high density and high incidence expression of GRPR have been documented in human PCa, especially in the early stages of the disease. Furthermore, the lack of GRPR expression in prostate hyperplasia offers the unique possibility to discriminate early neoplastic events from common hyperplasia [13,14]. The introduction of a promising and safe radiotracer for assessing GRPR-expression in PCa may serve as an alternative or complement to PSMA-directed probes. Implementation of the radiotheranostic concept in human PCa starts with diagnostic imaging to identify GRPR-positive lesions, which are amenable to treatment with a therapeutic anti-GRPR counterpart [15,16,17].

The search for GRPR-seeking radiotheranostic agents has been quite intense in recent years. The first peptidic analogs were based on the amphibian tetradecapeptide bombesin (BBN) and its C-terminal octa/nonapeptide fragments, retaining full capacity of interaction with the GRPR [18]. A great variety of peptides carrying various radiometal–chelate and linker combinations have been synthesized and evaluated in animal models, and a few have been tested in the clinic. Two major inherent shortcomings in the use of these peptides have led to concerns about the feasibility of this whole endeavor. On the one hand, acute adverse effects elicited by the activation of the GRPR after the intravenous injection of potent agonists in patients impose serious safety concerns [19,20]. On the other hand, the rapid hydrolytic breakdown of radioligands originating from native peptide motifs by omnipresent proteases compromises tumor targeting efficacy [18].

The first challenge has been elegantly addressed by shifting from GRPR agonists to antagonists, which do not activate the GRPR after binding [18,21]. Several studies on animals and later in men have shown the soundness of the GRPR-antagonist-targeting approach and emphasized the impact of the proper design of radiometal-carriers to improve diagnostic and therapeutic outcomes [18]. Additional benefits became unexpectedly apparent during the application of antagonists. Firstly, antagonists should allow for a safe increase in the injected peptide mass to selectively saturate GRPR populations in normal tissues while leaving most GRPRs in tumors unaffected. The higher permitted injected peptide masses reduce the need for superhigh molar activity. Secondly, GRPR antagonists have turned out to be more stable in vivo, presumably by being more exotic to the body compared with agonists, based more on native sequences [22]. Furthermore, radioantagonists have shown the tendency to clear more rapidly from physiological tissues (including the GRPR-rich pancreas and gastrointestinal tract, GIT) than from tumors when compared to agonists [18]. As a result, tumor-to-background ratios could be increased in the case of radiolabeled GRPR-antagonists.

Development of a GRPR-targeting agent suitable for SPECT should allow more patients to receive a timely diagnostic procedure and benefit from earlier PCa detection and staging. The GRPR antagonist [^99m^Tc]Tc-DB8 in this study carries an acyclic tetraamine chelator at the N-terminus (sequences of different BBN analogues are listed in Table S1). A stable octahedral *trans*-[^99m^Tc][Tc(V)(O)_2_(N_4_)]^+^ radiometal–chelate is formed during labeling, in high yield and purity at molar activities well suited for targeting high-affinity–low-capacity systems, such as GRPRs in tumors [23,24]. This radiometal–chelate complex is hydrophilic, favoring the peptide’s rapid excretion via the kidneys and the urinary system. This general pattern, originally observed in mice, was replicated in PCa patients: the GRPR agonist [^99m^Tc]Tc-DB4, carrying a similar N_4_ chelate, showed a low abdominal background, facilitating the detection of pathologic lesions in the gut [25]. This was a significant improvement in pharmacokinetics compared with the first clinically tested GRPR agonist, [^99m^Tc]Tc-RP527, which carried instead a neutral square pyramidal [^99m^Tc][Tc(V)O(N_3_S)] radiometal–chelate, leading to excessive hepatobiliary excretion, which impeded the clear visualization of abdominal lesions [26,27]. A recent study evaluating the GRPR-antagonist-based [^99m^Tc]Tc-maSSS-PEG_2_-RM26, carrying a similar radiometal–chelate to [^99m^Tc]Tc-RP527, reported excessive radioactivity accumulation in the gut, compromising image quality [28]. Preliminary studies with the GRPR-antagonist-based [^99m^Tc]Tc-DB15 (a Sar^11^-substituted analog of [^99m^Tc]Tc-DB8) in two breast cancer (BCa) patients [29] and [^99m^Tc]Tc-N4-BTG in four PCa patients with minimal biochemical recurrence [30] further confirmed the favorable pharmacokinetics conveyed by the *trans*-[^99m^Tc][Tc(V)(O)_2_(N_4_)]^+^ radiometal–chelate. However, these studies were conducted with a minimal amount of injected peptide, without any attempt to investigate the impact of peptide mass on diagnostic accuracy. In none of the above clinical studies was a mass escalation performed, but the injected peptide mass remained within the 12.5–40 μg range.

Recently, encouraging results from a Phase I study for [^99m^Tc]Tc-DB8 in female patients with breast cancer (BCa) were reported [31]. However, the known gender differences in muscle and bone mass, physiology, and hormone status could influence the biodistribution, excretion pathway, and rate, thus affecting dosimetry. Additionally, different tumor characteristics between PCa and BCa, including GRPR expression density and heterogeneity, and the level of GRPR expression in physiological tissues, might affect the imaging efficacy of radiopharmaceuticals, like [^99m^Tc]Tc-DB8. In the present study, we first investigated the performance of [^99m^Tc]Tc-DB8 in male patients with PCa, collecting new information on GRPR-expression levels in PCa, assessing the tolerability and safety of the agent, and evaluating biodistribution and dosimetry calculations in men. We next performed a mass escalation study for the first time on PCa patients, aiming to improve tumor-to-background contrast and, thereby, to enhance diagnostic accuracy.

## 2. Materials and Methods

### 2.1. Study Design

This Phase I clinical trial was conducted as a single-center diagnostic open-label exploratory study at the Cancer Research Institute, Tomsk National Research Medical Center of the Russian Academy of Sciences (ClinicalTrials.gov ID NCT05940298). The initial study protocol was approved by the Institute’s Scientific Council and Board of Medical Ethics (protocol 7, approved 16 January 2023).

The single intravenous injections of [^99m^Tc]Tc-DB8 (labeled according to a published protocol [31]) were performed as a 10 mL solution of 40, 80, or 120 µg of peptide in saline within 1 min. Patients underwent whole-body planar imaging (anterior and posterior) 2, 4, 6, and 24 h post-injection (pi) and SPECT-CT acquisition 2, 4, and 6 h pi. The vital functions were analyzed before and 1 d after injection. The appearance of side effects was monitored for up to 7 d.

### 2.2. Patients

**Figure 1:**
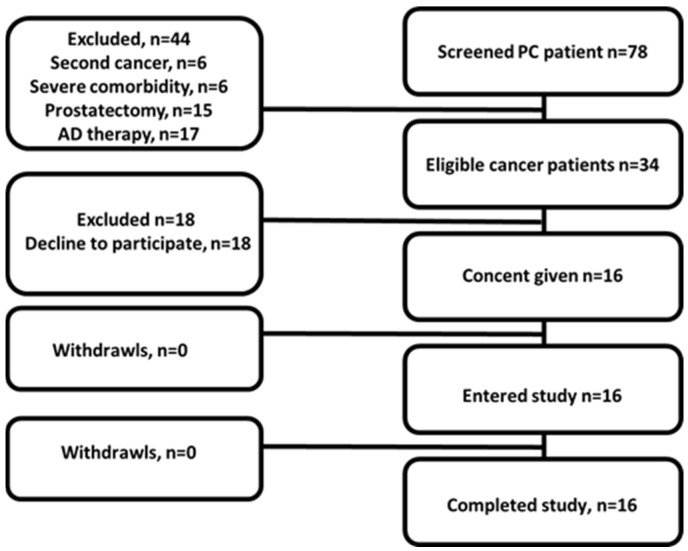
Flow diagram according to Standards for Reporting of Diagnostic Accuracy Studies for PCa patients.

Sixteen male patients (age 67 ± 7 y, 48 to 76 y) with clinically and radiologically diagnosed and histologically verified hormone-sensitive or castration-resistant PCa were included in this study after providing written informed consent (Figure 1). A pre-imaging diagnostic examination of patients included a measurement of PSA levels, TRUS examination, MRI investigation of the pelvic organs with contrast, chest CT, bone scans using [^99m^Tc]Tc-pyrophosphate, and histopathological examination of PCa biopsy material.

Criteria for inclusion were as follows: histologically verified PCa; high probability of distant metastases; the ability to undergo planned diagnostic investigations; and the absence of hematological, hepatic, and renal pathologies. The exclusion criteria were as follows: ECOG (Eastern Cooperative Oncology Group) degree > 2; CKD (chronic kidney disease) stage 4 or higher; hepatitis B or C, HIV, or infectious diseases within the preceding 3 months; participation in other clinical studies within 30 days; and a severe somatic condition caused by concomitant pathology or underlying disease.

### 2.3. SPECT Imaging Protocol

Imaging was performed using a hybrid system, Symbia Intevo Bold (Siemens, Forchheim, Germany), equipped with a dual-head gamma camera and an integrated 16-slice CT scanner. A low-energy, high-resolution collimator was used. Patients underwent whole-body planar imaging (anterior and posterior, a scan speed of 12 cm/min, 1024 × 256 pixel matrix), SPECT-CT acquisition (32 projections, 30 s each, 128 × 128 pixel matrix), and a low-dose CT (140 kVp, 20 mAs/slice, 512 × 512 pixel matrix). The syngo.via workstation (Siemens, Forchheim, Germany) was used for the image analysis. Visualization of PSMA expression in Patient 10 was performed using [^99m^Tc]Tc-BQ0413 (see study protocol in [32]) 1 month before this study.

### 2.4. Assessment of Dosimetry

The activity distribution in organs estimated from regions of interest (ROIs) over time (4 time points) was used to calculate average residence times by fitting them to a single exponential function using Prism 10.4.0 (GraphPad Software, LLC, Boston, MA, USA), as previously reported [31]. Absorbed doses for individual organs, effective doses, and effective dose equivalents were calculated in OLINDA/EXM 1.1 using an adult male phantom.

### 2.5. GRPR Expression Analysis by Immunohistochemistry

GRPR expression was evaluated using automated immunohistochemistry on the Leica Bond Max platform (Leica Biosystems, Nussloch, Germany). Tissue sections (biopsies before any treatment) were stained with a monoclonal anti-GRPR antibody (clone 18H31L38, Cat. No. 703928, Thermo Fisher Scientific, Waltham, MA, USA) at a 1:500 dilution, with appropriate positive (pancreas tissue) and negative (lymph node) controls included in each run. Stained slides were digitally scanned at ×40 magnification using the Aperio AT2 system (Leica Biosystems, Nussloch, Germany) for subsequent analysis.

A senior pathologist performed a blinded evaluation of GRPR expression using a standardized immunoreactive score (IRS) system [33]. This semi-quantitative assessment incorporated two parameters: (1) staining intensity (graded as 0 = absent, 1 = weak, 2 = moderate, 3 = strong) and (2) percentage of positive tumor cells (0 = 0%, 1 ≤ 10%, 2 = 11–50%, 3 = 51–80%, 4 > 80%). The final IRS (range, 0–12) was calculated by multiplying the intensity score by the percentage score.

### 2.6. Statistical Analysis

Data are reported as means with standard deviations (n = 5–6). The median and interquartile ranges, Me [Q1–Q3], were used to present nonparametric data. Differences in significance (one-way ANOVA, two-sided, *p* < 0.05) were tested using Prism 10.4.0 (GraphPad Software, LLC, Boston, MA, USA).

## 3. Results

### 3.1. Patients

All patients included in the study were diagnosed with prostatic acinar adenocarcinoma G1-G5, GS 6-10, and median PSA 11.4 ng/mL [0.19-800] (Table 1). One patient underwent prostatectomy but showed recurrence according to MR imaging. Twelve patients had clinically proven metastases: six patients had lymph node metastases (LNMs), and six patients had multiple LNMs and bone metastases (BMs).

### 3.2. Safety, Tolerability, and Activity Distribution of [^99m^Tc]Tc-DB8

The safety and tolerability of single intravenous administration of [^99m^Tc]Tc-DB8 were confirmed by the absence of any complaints from patients or any adverse events, vital sign changes, or abnormal clinical laboratory findings for all tested peptide masses injected.

**Figure 2:**
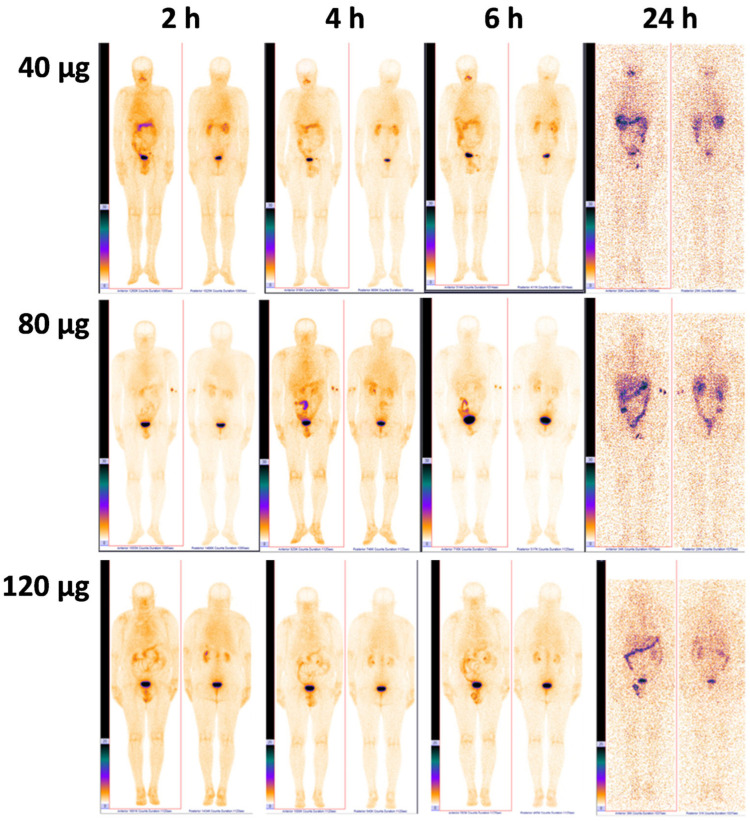
Representative anterior and posterior images of PCa patients 2, 4, 6, and 24 h pi of [^99m^Tc]Tc-DB8 corresponding to 40, 80, and 120 µg of peptide mass. A linear relative scale (normalized at the maximum activity in the image) is applied.

The activity distribution in PCa patients after administration of [^99m^Tc]Tc-DB8 at different peptide doses (40 μg, 80 μg, and 120 μg) did not differ visually on planar images (Figure 2). At 2 h after administration of the radioligand, its accumulation was already noted in the pancreas, small intestine, kidneys, and urinary bladder. After 24 h, accumulation was visualized along the colon. Activity accumulation in anatomically significant organs—background organs for imaging PCa and its early metastases—was significantly lower than in the organs of the urinary system and organs with endogenous expression of the GRPR. The pharmacokinetics of [^99m^Tc]Tc-DB8 were significantly different in the patient with a massive BM (Patient 8) than in other patients. The labeled peptide had already accumulated predominantly in the bone metastases 2 h pi, and this accumulation persisted for up to 24 h during the study without significant redistribution (Figure S1).

**Figure 3:**
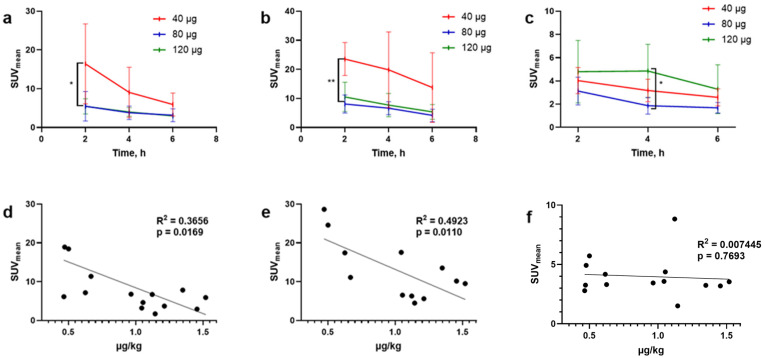
Activity uptake over time in kidneys (**a**), pancreas (**b**), and primary tumors (**c**) in PCa patients after a single injection of [^99m^Tc]Tc-DB8 (presented as an average SUV value for the group, SUV_mean_). The activity uptake was significantly higher for patients injected with 40 µg of the peptide at 2 h pi; for kidneys, *p* < 0.05 (*), and for pancreatic tissue, *p* < 0.001 (**). Dependence of activity uptake in kidneys (**d**), pancreas (**e**), and primary tumors (**f**) on injected peptide mass (presented as µg of peptide per kg body weight of individual patient) 2 h pi of [^99m^Tc]Tc-DB8.

**Figure 4:**
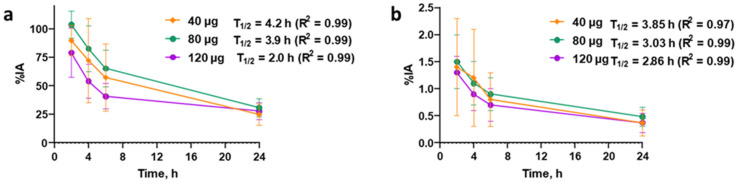
Decay-corrected kinetics of whole-body elimination (**a**) and blood clearance (**b**) of [^99m^Tc]Tc-DB8 depending on injected peptide mass. Data are taken from whole-body planar images.

The overall activity distribution pattern was characterized by rapid activity excretion, predominantly via the kidneys. The activity uptake in kidneys and pancreatic tissue was higher (significantly higher at 2 h pi) in the patients injected with 40 µg of peptide than in the patients injected with 80 or 120 µg (Figure 3a,b). The pancreas was well visualized, regardless of the dose of peptide administered (Figure S2). Uptake in the kidneys decreased 2–3-fold between 2 and 6 h pi, and the activity from the kidneys was washed out in the urine. Activity uptake in other normal tissues was low. An increase in injected peptide mass led to a tendency toward faster whole-body activity elimination and blood clearance of [^99m^Tc]Tc-DB8 (Figure 4).

### 3.3. Dosimetry Assessment

The absorbed doses in normal organs were estimated using planar whole-body images after injection of three different masses of peptide (Table 2). The absorbed doses demonstrated a tendency to decrease with an increase in injected peptide mass. However, a significantly higher absorbed dose after the injection of 40 µg compared to that for 120 µg was found only for the pancreas. The effective doses for different peptide masses did not differ significantly and were in the range 0.0038–0.0066 mSv/MBq, which corresponded to 1.6–3.0 mSv/procedure.

### 3.4. Imaging Findings

**Figure 5:**
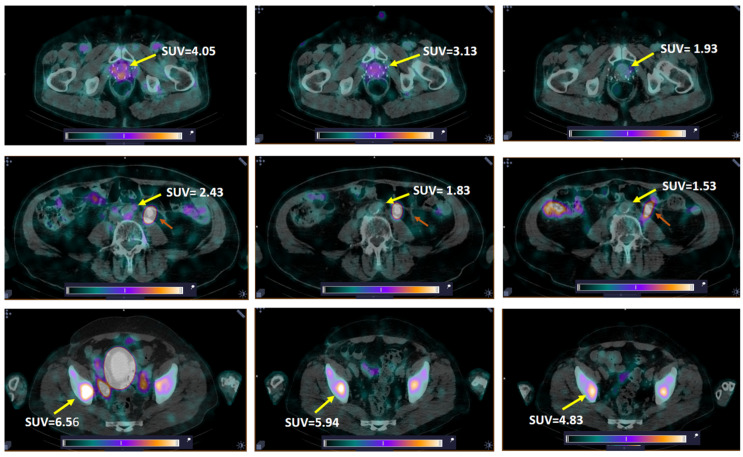
Decrease in tissue uptake (SUV_max_) in primary tumor (**upper panel**, Patient 7), LNM (**middle panel**, Patient 13, paraaortic lymph node), and BM (**bottom panel**, Patient 10) with time. Yellow arrow—lesion; red arrow (**middle panel**)—dilated left ureter. A linear relative scale (normalized at the maximum activity in the image, SUV_max,_ from 0 to 5.0) is applied.

High-contrast images of tumors were obtained 2 h after administration of [^99m^Tc]Tc-DB8 for all tested masses of peptide. Primary PCa lesions were visualized in 14 of 15 patients with primary tumors (93.3%; 1 patient underwent prostatectomy). The activity uptake in primary tumor, LNM, and BM lesions had a tendency to decrease with time (Figure 3c and Figure 5).

**Figure 6:**
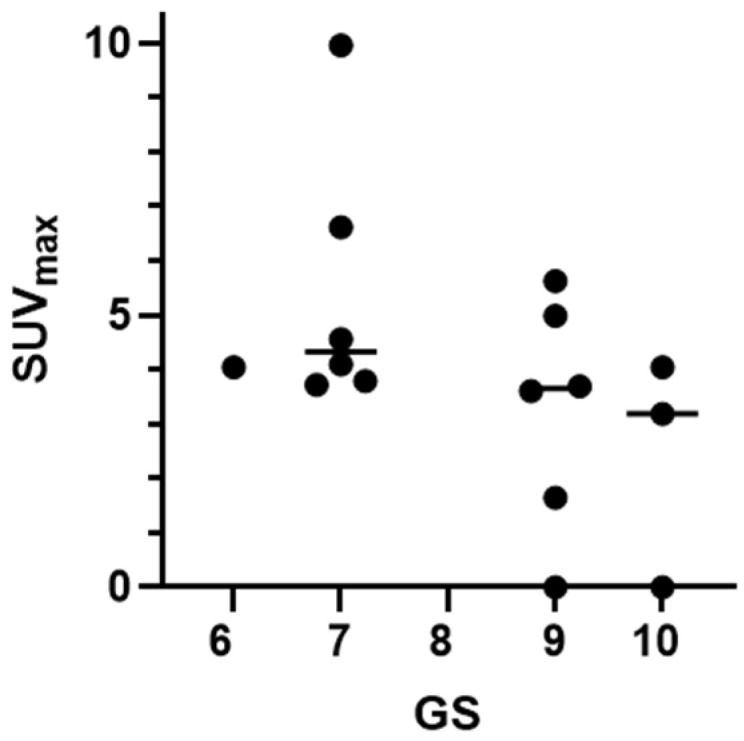
Activity uptake in primary tumors (SUV_max_) 2 h pi of [^99m^Tc]Tc-DB8 (pooled data), depending on histopathologically determined GS, demonstrated a tendency to decrease with GS increase.

The activity uptake in primary lesions has a tendency to be higher after administration of [^99m^Tc]Tc-DB8, corresponding to 120 µg peptide mass; however, the difference was statistically significant only at 4 h pi between uptakes after injections of 120 µg and 80 µg (Figure 3c). The activity uptake in primary lesions did not correlate with PSA concentrations in the blood, tumor size, or castration resistance. However, there was a tendency toward lower tumor uptake with increasing GS, determined by pathology examination (Figure 6). The image contrast (tumor-to-surrounding tissue SUV_mean_ ratios) increased with time, a finding that was more pronounced for the group injected with the lowest peptide mass (Table 3).

**Figure 7:**
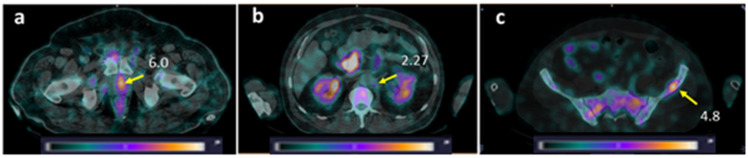
SPECT/CT images of PCa patients 2 h after injection of [^99m^Tc]Tc-DB8. The foci of increased [^99m^Tc]Tc-DB8 uptake (yellow arrow, SUV_max_) are visualized (**a**) in the prostate (Patient 16); (**b**) in the paraaortic lymph node on the left (Patient 10); and (**c**) in the left ilium (Patient 10). A linear relative scale (normalized at the maximum activity in the image SUV_max_ from 0 to 5.0) is applied.

LNMs were visualized in 2 out of 12 patients with known lesions (16.7%), and BMs in 2 out of 6 patients with known lesions (33.3%) (Figure 7). Additionally, activity uptake in BMs was visualized in two patients without known bone involvement according to CT. Patients with visualized LNMs and BMs were administered 80 or 120 µg of peptide. The SUV_max_ values for LNMs and surrounding tissues for individual patients were lower than for primary tumors in both groups injected with 80 and 120 µg of peptide, while for BMs, the group injected with 80 µg had a higher SUV_max_ than for primary tumors (Table 1). Nevertheless, the ratios for SUV_mean_ in metastatic lesions to surrounding tissues were suitable for visualization: for LNM, 1.97–3.49 for 80 µg and 2.40–2.61 for 120 µg; for BM, 3.97–9.81 and 1.72–2.53, respectively.

**Figure 8:**
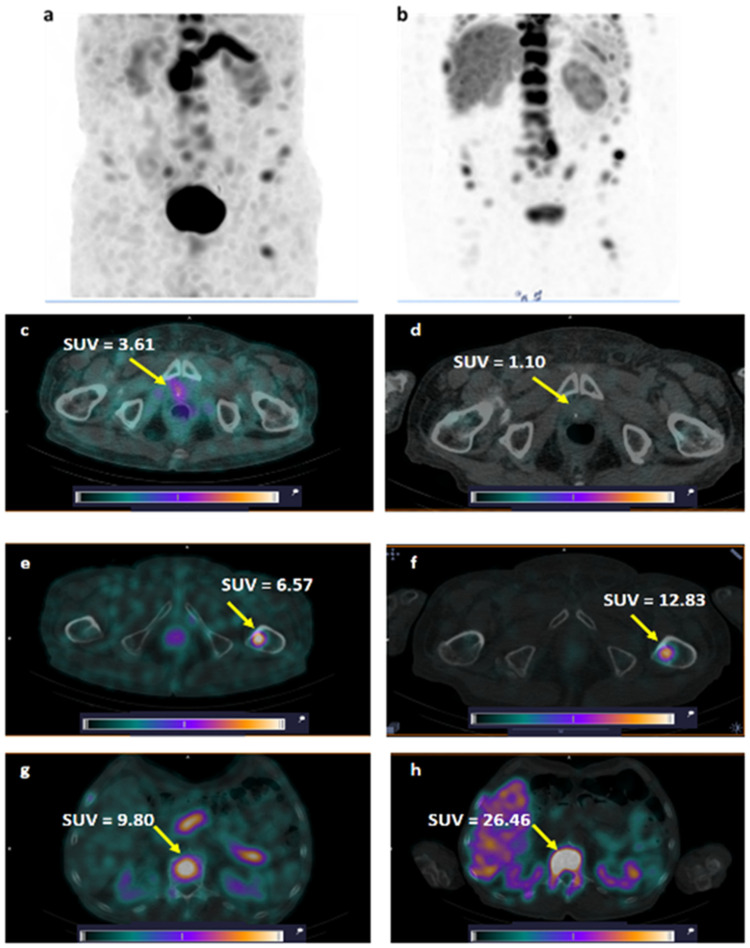
Comparison of visualization of GRPR expression using [^99m^Tc]Tc-DB8 (**a**) and PSMA expression using [^99m^Tc]Tc-BQ0413 (**b**) in primary tumor (**c**,**d**) and BM (**e**–**h**) in Patient 8. A linear relative scale (normalized at the maximum activity in the image SUV_max_ from 0 to 5.0) is applied.

One patient (Patient 8, injected with a peptide mass of 80 µg) underwent SPECT visualization of PCa using the anti-PSMA targeting agent [^99m^Tc]Tc-BQ0413 1 month before the current study (see Phase I study report [32], Patient 5). The comparison of GRPR SPECT and PSMA SPECT (Figure 8) revealed higher tumor uptake for [^99m^Tc]Tc-DB8 in the primary lesion in the prostate gland. BM lesions were visualized with both agents; however, the contrast was higher for PSMA SPECT.

### 3.5. Immunohistochemistry

**Figure 9:**
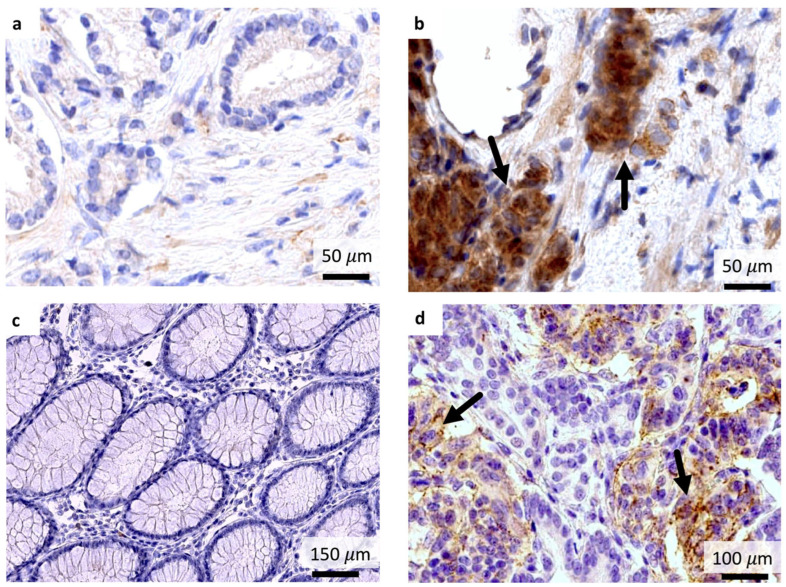
Interpersonal heterogeneity of GRPR expression in PCa tissue. Weak GRPR expression, IRS 1, and SUV_max_ 3.72 on SPECT images 2 h pi, Patient 2 (**a**); strong GRPR expression, IRS 8, and SUV_max_ 5.63, Patient 1 (**b**). Intestinal tissue was used as a negative staining control (**c**), and pancreatic tissue was used as a positive staining control (**d**). Strong membranous/cytoplasmic expression is indicated by black arrows. Magnification ×100–200.

Evaluation of GRPR expression in available patient biopsy samples (n = 6, Table 1) revealed pronounced intertumoral expression heterogeneity (Figure 9). Formal correlation analysis between GRPR immunohistochemical scores (IRS) and tumor uptake (SUV_max_) on SPECT imaging could not be performed due to variability in administered doses across patients. No correlation was observed between GRPR expression in biopsy samples and GS, PSA, and PAA G_x_ (R^2^ = 0.56, *p* = 0.30; R^2^ = 0.42, *p* = 0.46; and R^2^ = 0.06, *p* = 0.93, respectively).

## 4. Discussion

The visualization of GRPR expression was proposed for diagnosing PCa as early as thirty years ago, and in 2000, the first peptide radioligand, [^99m^Tc]Tc-RP527, was clinically tested in PCa patients [26]. Further development of GRPR-agonist-based radioligands focused on stabilization of peptides to proteolytic degradation, improvement of radiolabel stability, and overall efforts to increase hydrophilicity to minimize hepatobiliary excretion [18]. This led to the development of [^99m^Tc]Tc-DB4, already demonstrating high contrast visualization of GRPR expression in PCa patients at 1 h pi [25]. However, gastrointestinal adverse events, such as nausea, vomiting, diarrhea, constipation, and dysgeusia, were observed in patients injected with as low as 12 µg of peptide, although these effects could not be unequivocally attributed to the peptide injection. Likewise, severe side effects provoked by GRPR activation prevented the clinical use of radiolabeled GRPR agonists for imaging and especially therapy of tumors [19].

Two major factors played a significant role in revitalizing interest in introducing GRPR-based diagnostics for PCa: Firstly, the development of radioligands occurs based on GRPR antagonists, which do not trigger side effects and do not provoke GRPR downregulation since they do not activate the GRPR [21,34,35,36,37]. The last-mentioned effect leads to improved tumor targeting [37]. Secondly, the suboptimal expression of PSMA, the most utilized molecular target in PCa, occurs in 30–40% of PCa [38].

The development of GRPR-targeting peptides suitable for PET diagnosis has been the main focus within the last few years (see a recent review [39]). They have demonstrated great sensitivity for localized low-risk PCa, where GRPR expression is usually higher than the expression of PSMA [17]. The search for GRPR-antagonist-based radioligands for SPECT imaging has been less active. So far, only four peptides labeled with Tc-99m have been tested clinically: [^99m^Tc]Tc-DB15 in BCa patients [29], [^99m^Tc]Tc-maSSS-RM26 in PCa and BCa patients [28], and [^99m^Tc]Tc-N4-TBG in PCa patients with biochemical recurrence [30]. Recently, our group presented results on imaging GRPR in BCa patients using the GRPR antagonist [^99m^Tc]Tc-DB8 [31]. The administration of [^99m^Tc]Tc-DB8 was well tolerated. All primary tumors and all known LNMs were visualized, and the activity uptake was found to be higher in estrogen-positive lesions. The remarkable finding of that study was the observation that the injected peptide mass had a significant impact on tumor activity uptake and imaging contrast.

We report herein the results of a Phase I clinical study on the visualization of GRPR expression in male PCa patients using the new GRPR antagonist [^99m^Tc]Tc-DB8, suitable for SPECT, which demonstrated a biodistribution profile suitable for GRPR visualization in preclinical studies in PCa models in mice [23]. The development of a GRPR-targeting imaging probe could improve the diagnosis of PCa with insufficient PSMA expression, while the use of the SPECT imaging modality could make this diagnostic procedure available to a wider patient population [16].

Like the study on BCa patients, this study demonstrated that [^99m^Tc]Tc-DB8 is safe and well tolerated in PCa patients [31]. It should be highlighted that the highest injected peptide mass in this study, 120 µg, was 10-fold higher than the injected mass in the case of [^99m^Tc]Tc-DB4, a potent agonist that triggers adverse events [25], and at least 2-fold higher than for other clinically tested antagonists (the highest reported peptide mass was 56 µg for [^68^Ga]Ga-RM2 [40]). The absence of side effects and changes in vital parameters in all patients allowed us to further optimize the injected peptide mass to obtain a higher contrast image of GRPR expression in PCa patients.

The highest absorbed dose per organ was found for the pancreas. Pancreatic tissue shows high GRPR expression, including expression in the islets, which is important for the normal regulation of islet function to mediate GRP-induced insulin secretion and results in high uptake of GRPR-targeting peptides [41,42]. High activity uptake in pancreatic tissue could interfere with the detection of LNMs close to this organ; however, we have demonstrated in this study that activity uptake in pancreatic tissue could be partially saturated if a higher peptide mass was injected (Figure 3b).

Elevated absorbed doses were found for excretory organs (kidneys with urinary bladder wall and gastrointestinal tract wall), the thymus, and osteogenic cells (Table 2). The absorbed doses for individual organs and the effective dose demonstrated a tendency to decrease with increasing injected peptide mass, in agreement with the faster clearance of activity for a higher injected peptide mass (Figure 4). The dose for the kidneys was significantly lower for the administration of 120 µg than for 40 µg of the peptide. It should be noted that in patients with massive BM lesions, the radiation doses to organs and tissues may differ from those calculated for patients with localized PCa. In the only such case included in our study, [^99m^Tc]Tc-DB8 was stably taken up by the BM without any sign of redistribution (Figure S1). The level of drug accumulation in the BM was comparable to the level of accumulation in critical organs (kidneys, urinary bladder, and pancreas). To calculate radiation doses for such patients, additional studies are needed in a larger cohort of patients with widespread metastases.

The overall distribution of [^99m^Tc]Tc-DB8 in PCa patients (males) was generally similar to that in BCa patients (females) [31], but in the PCa patients, hepatobiliary excretion was lower. This difference resulted in 1.1–1.5-fold-lower absorbed doses in organs of the gastrointestinal tract for male PCa patients. The decreased hepatobiliary excretion in male patients was associated with a 2-fold-higher absorbed dose for kidneys, 0.014 ± 0.005 mGy/MBq vs. 0.007 ± 0.002 mGy/MBq for female patients (both values for the injection of 80 µg of peptide). A similar phenomenon of a higher degree of hepatobiliary excretion in female patients was observed for other GRPR-targeting peptides, [^68^Ga]Ga-NOTA-RM26 and [^99m^Tc]Tc-maSSS-RM26 [28,43,44]. This could be explained by the slower clearance rate for GRPR-targeting peptides in females, as observed in our previous study on [^99m^Tc]Tc-maSSS-RM26 [28]. The rate of hepatobiliary/renal excretion, to a certain extent, is influenced by physiological differences between genders, e.g., hepatic blood flow, renal blood flow, glomerular filtration, tubular secretion, and tubular reabsorption—all slower in women than in men [45].

The comparison of the absorbed doses for organs and the effective dose of [^99m^Tc]Tc-DB8 for PCa patients with those reported for [^99m^Tc]Tc-labeled GRPR-targeting peptides [^99m^Tc]Tc-maSSS-RM26 and [^99m^Tc]Tc-N4-BTG revealed a similar pattern, with elevated doses for pancreatic tissue, the kidneys, and the urinary bladder wall [28,30]. The absorbed doses for healthy organs and the effective dose for a single injection of [^99m^Tc]Tc-DB8 were similar to values for routinely clinically used bone scintigraphy [46].

The detection rate for primary PCa lesions was over 90%. The imaging contrast was high both for primary and metastatic lesions (uptake in primary lesion to uptake in surrounding tissue, Table 3). The patient with a non-visualized tumor had advanced disease, a PSA level in blood of 418 ng/mL, and GS 10 (Patient 14). However, a local recurrence was visualized with high activity uptake in the lesion for the patient with a long history of disease (SUV_max_ 6.62) and with evidence of minimal biochemical recurrence (PSA = 0.27 ng/mL) (Patient 3). Only a few LNMs and BMs were visualized in this cohort despite the inclusion criteria, e.g., high probability of LNM, and presence of BMs according to osteoscintigraphy.

All patients with visualized LNMs and BMs were injected with 80 or 120 µg of peptide. The activity uptake (SUV_max_) in LNMs and BMs after injection of 120 and 80 µg at 2 h pi for all tested peptide doses did not differ significantly. The activity uptake in the lesions decreased with time; however, the imaging contrast increased due to faster clearance of activity from blood and healthy tissue (Figure 5 and Table 3). The comparison of activity uptake in primary tumors and uptake in the kidneys and pancreas (organs with the highest activity uptake in the abdomen) clearly demonstrated that activity uptake in healthy tissue decreased with an increase in the injected peptide mass, while uptake in primary PCa tumors did not differ significantly (Figure 3d–f). This could be tentatively attributed to differences in peptide delivery and uptake by the target cells, as well as subsequent activity release thereof between physiological tissues and tumors as a result of several distinct factors. Such factors are related to differences in GRPR expression patterns, anatomical location, and vascularization, as well as the overall biochemical milieu around the GRPR target area (especially in terms of local enzyme activity). It is not yet fully understood how these factors lead to the observed differences in uptake and retention between healthy tissues and tumors expressing the target. Nevertheless, similar effects have consistently been reported for other cancer-associated molecular targets, e.g., somatostatin receptors [47], PSMA [48], and human epidermal growth factor receptors type 1 [49] and type 2 [50]. In contrast, both activity uptake in the lesions and tumor-to-background ratios were significantly higher for the intermediate peptide dose of 80 µg in female patients with BCa [31]. It might be speculated that this difference could be related to a difference in GRPR expression between PCa and BCa, on the one hand, and, on the other, to gender. Thus, the use of a higher peptide dose should enhance high-contrast imaging in PCa. Imaging at 2–4 h pi of 80–120 µg of [^99m^Tc]Tc-DB8 could be recommended for future clinical studies based on activity uptake in lesions and in healthy tissues observed in this study.

A promising feature of [^99m^Tc]Tc-DB8 may derive from its high uptake in the pancreas, associated with GRPR expression on islet cells. In our study, the pancreas was well visualized in all patients, regardless of the dose of peptide administered (Figure S2). There are currently no approved radiopharmaceuticals for pancreatic examination in nuclear medicine, and [^99m^Tc]Tc-DB8 may serve as one. It is known that changes in GRPR expression are observed in pancreatitis and pancreatic cancer [51]; thus, a further promising area of [^99m^Tc]Tc-DB8 imaging applications may be the assessment of pancreatic transplant viability. Additional studies are necessary in patients with pancreatic pathology to assess the diagnostic significance of pancreatic scintigraphy with [^99m^Tc]Tc-DB8 and to determine the optimal dose of the peptide and the timing of SPECT/CT examination.

Comparison with other reported peptides for SPECT visualization of GRPR expression in PCa is difficult and cannot be straightforward. Both [^99m^Tc]Tc-N4-BTG and [^99m^Tc]Tc-maSSS-RM26 [29,31] were injected in suboptimal peptide masses (12 µg and 40 µg, respectively). No lesions suggestive of PCa were visualized when [^99m^Tc]Tc-N4-BTG was used. [^99m^Tc]Tc-maSSS-RM26 visualized four of six (66.6%) primary tumor lesions (SUV_max_, ~ 1.2, and tumor–background (muscle) ratio, ~6.9, 2 h pi) but none of the known LNM and BM lesions. For [^99m^Tc]Tc-DB8 at the same time point, the detection rates for primary lesions, LNMs, and BMs were higher (93.0%, 16.7%, and 33.3%, respectively), and so was activity accumulation in primary lesions (SUV_max_ 4–5). These differences could be explained by other factors besides simply the better performance of [^99m^Tc]Tc-DB8. The present study has demonstrated that a much higher peptide injected mass is required for higher lesion-to-healthy tissue ratios without posing any biosafety concerns. Furthermore, the differences in patient selection and relatively small cohorts, common in Phase I trials, should also be taken into account. Patients with biochemical recurrence of PCa were selected for imaging with [^99m^Tc]Tc-N4-BTG, while mainly newly diagnosed-with-PCa patients were selected for Phase I studies with [^99m^Tc]Tc-maSSS-RM26 and [^99m^Tc]Tc-DB8. Compelling evidence points to the higher accuracy of GRPR-imaging peptides in the detection of low- and intermediate-risk PCa, with lower PSA levels, GS, clinical stages, and EAU-risk categories [39,52]. This is in good agreement with the expression pattern of the GRPR in PCa: the GRPR expression rate and density decrease with disease progression [53,54]. In fact, the tendency of a decrease in activity uptake with an increase in GS was evident even in this study with relatively small patient cohorts (Figure 6).

[^99m^Tc]Tc-DB8, a GRPR-imaging SPECT tracer, demonstrated a high detection rate for primary PCa lesions similar to PET tracers [^68^Ga]Ga-RM2, [^68^Ga]Ga-SB3, [^68^Ga]Ga-BAY-7548, and [^68^Ga]Ga-NOTA-RM26, 84–89% [44,51,52,53,55,56,57]. In patients with biochemically recurrent PCa, the reported sensitivity for [^68^Ga]Ga-RM2 and [^68^Ga]Ga-NOTA-RM26 was lower, by 60–70% [52,58]. This might be a strong indication that GRPR imaging could be more effective in newly diagnosed therapy-naïve patients and patients with oligometastatic disease.

The comparison of GRPR and PSMA diagnostic images in Patient 8 (GS 9, PSA 800 ng/mL) demonstrated that the primary lesion in the prostate gland was clearly visualized only with the GPRP-targeting [^99m^Tc]Tc-DB8 (SUV_max_ 3.61) but not with the PSMA-targeting [^99m^Tc]Tc-BQ0413 (SUV_max_ 1.10). Conversely, LNMs and BMs had higher uptake in the case of [^99m^Tc]Tc-BQ0413 (Figure 8). These results are in good agreement with other published clinical data when GRPR and PSMA diagnostic imaging peptides were head-to-head compared in large cohorts of PCa patients [8,9,58,59].

The main limitation of this study was the small number of patients included in each group (5–6 patients). In addition, the groups were heterogeneous with respect to the hormone sensitivity of PCa: in the first group (40 µg), all patients were hormone-sensitive; in the second group (80 µg), hormone-sensitive patients predominated; and in the third group (120 µg), castration-resistant patients constituted the majority. There was also heterogeneity in the presence of distant metastases of PCa: in the first group (40 µg), only one patient was diagnosed with M1, while in the third group (120 µg), stage M1 was found in the majority of patients. Another limitation of the study was the small number of biopsy material available for GRPR expression determination by IHC (five patients). All these limitations should be taken into account during the planning of the second phase of clinical trials.

## 5. Conclusions

Single intravenous administration of [^99m^Tc]Tc-DB8 for visualization of GRPR expression in PCa using SPECT imaging was well tolerated in a peptide mass range of 40–120 µg. An increase in the injected mass resulted in 3-fold-lower activity uptake in the pancreas, an organ with endogenous GRPR expression, and 2.5-fold-lower activity uptake in the kidneys. Primary PCa lesions were already clearly visualized 2 h pi in 14 of 15 PCa patients under all injected peptide mass regimens tested. An injected peptide mass of 80–120 µg/patient and SPECT acquisition 2–4 h pi were found to be optimal for further clinical studies due to the significantly lower activity accumulation in the pancreas and kidneys. The results of this study are expected to be of interest in the nuclear medicine and oncology communities, especially when taking into account the excellent nuclear properties of Tc-99m for SPECT imaging, the continuing wide availability of SPECT cameras, and related recent technological advancements.

## Figures and Tables

**Table 1:** Patient characteristics and imaging findings in patients administered with [^99m^Tc]Tc-DB8.

Dose (µg)	Patient/ Age (y)	Weight (kg)	Histotype Grade Group/Gleason Score/PSA * (ng/mL)	Clinical Stage	MRI Size (mm)	Imaging Results 2 h pi (SUV_max_)		IRS
						PT	LNM and BM	
40	1/73	84	PAA G5GS 9 (4 + 5) PSA = 34.5	T3N1M1 HS	39 × 22	5.63	LNM—N/D BM—N/D	(2 × 4) 8
	2/66	85	PAA G2GS 7 (3 + 4) PSA = 5.4	T2N0M0 HS	8 × 9	3.72	LNM—N/D BM—N/D	(1 × 1) 1
	3/73	80	PAA G3GS 7 (4 + 3) PSA = 0.27 Brachytherapy > 10 y (2011)	T2cNxM0 HS	41 × 50	6.62	LNM—N/D BM—N/D	
	4/74	65	PAA G2GS 7 (3 + 4) PSA = 1.07	T3bNxM0 HS	67 × 45	4.56	LNM—N/D BM—N/D	
	5/68	64	PAA G3GS 7 (4 + 3) PSA = 80.47	T2NxM0 HS	22 × 18	3.79	LNM—N/D BM—N/D	
	6/57	86	PAA G5GS 10 (5 + 5) PSA = 1.28	T4N1M0 HS	42 × 31	3.19	LNM—N/D BM—N/D	(2 × 4) 8
80	7/72	83	PAA G1GS 6 (3 + 3) PSA = 52	T2N0M0 HS	14 × 10	4.05	LNM—N/D BM—N/D	
	8/76	55	PAA G5GS 9 (4 + 5) PSA = 800	T3bNxM1 CR	16 × 6	3.61	LNM—N/D BM—9.8 ***	
	9/63	120	PAA G5GS 9 (4 + 5) PSA = 7.3 Prostatectomy > 10 y (2011)	T3N0M0 CR	50 × 25	N/D	LNM—N/D BM—N/D (*CT positive*)	
	10/65	76	PAA G5GS 9 (4 + 5) PSA = 100	T3N1M1 HS	41 × 36	4.99	LNM—2.27 (*Paraaortic*) BM—6.56	(1 × 4) 4
	11/48	70	PAA G5GS 9 (4 + 5) PSA = 0.19	T3bNxM0 HS	35 × 26	1.65	LNM—N/D BM—N/D (*CT positive*)	(1 × 3) 3
120	12/70	107	PAA G3GS 7 (4 + 3) PSA = 41	T3bN1M1 CR	19 × 11	9.96	LNM—N/D BM—N/D	
	13/72	79	PAA G5GS 10 (5 + 5) PSA = 11.21	T2aN1M1b CR	34 × 33	4.05	LNM—2.79 (Mesenteric) BM—N/D	
	1/604	99	PAA G5GS 10 (5 + 5) PSA = 418	T4N1M1 CR	22 × 31	N/D	LNM—N/D BM—N/D	
	15/64	89	PAA G5GS 9 (4 + 5) PSA = 11.4	T3bN1M1 CR	25 × 22	3.7	LNM—N/D BM—2.0	
	16/62	115	PAA G2GS 7 (3 + 4) ** PSA = 1.5	T1aN0M0 HS	37 × 28	6.0	LNM—N/D BM—2.25	(2 × 3) 6

* PAA—prostatic acinar adenocarcinoma; Gx—ISUP Grade Group; GS—Gleason score; PSA—prostate-specific antigen concentration in blood, ng/mL; HS—hormone sensitive; CR—castration-resistant; PT primary tumor; LNM—lymph node metastasis; BM—bone metastasis; N/D—not detected; IRS—immunoreactive score (determined immunohistochemically). ** PSA results 9 m old. *** In cases of multiple metastases, the highest values are given.

**Table 2:** Absorbed doses after the single-bolus administration of [^99m^Tc]Tc-DB8.

Organ	40 µg	80 µg	120 µg
Adrenals	0.0064 ± 0.0036	0.0060 ± 0.0001	0.0036 ± 0.0015
Brain	0.0012 ± 0.0006	0.0015 ± 0.0005	0.0009 ± 0.0003
Breasts	0.0011 ± 0.0005	0.0013 ± 0.0003	0.0008 ± 0.0002
Gall bladder wall	0.0049 ± 0.0014	0.0059 ± 0.0012	0.0048 ± 0.0025
LLI wall	0.0054 ± 0.0027	0.0077 ± 0.0035	0.0049 ± 0.0007
Small intestine wall	0.0049 ± 0.0024	0.0065 ± 0.0021	0.0039 ± 0.0008
Stomach wall	0.0040 ± 0.0019	0.0047 ± 0.0010	0.0030 ± 0.0011
ULI	0.0052 ± 0.0024	0.0060 ± 0.0017	0.0041 ± 0.0006
Heart wall	0.0043 ± 0.0022	0.0050 ± 0.0013	0.0034 ± 0.0008
Kidneys	0.0073 ± 0.0025	0.0066 ± 0.0016	0.0054 ± 0.0024
Liver	0.0037 ± 0.0018	0.0040 ± 0.0005	0.0029 ± 0.0007
Lungs	0.0030 ± 0.0013	0.0034 ± 0.0012	0.0023 ± 0.0006
Muscle	0.0019 ± 0.0009	0.0026 ± 0.0012	0.0015 ± 0.0004
Pancreas	0.0196 ± 0.0090 *	0.0119 ± 0.0034	0.0071 ± 0.0025
Red Marrow	0.0026 ± 0.0013	0.0035 ± 0.0015	0.0021 ± 0.0006
Osteogenic cells	0.0067 ± 0.0034	0.0092 ± 0.0040	0.0052 ± 0.0018
Skin	0.0015 ± 0.0008	0.0021 ± 0.0010	0.0012 ± 0.0004
Spleen	0.0051 ± 0.0023	0.0059 ± 0.0012	0.0046 ± 0.0017
Testes	0.0047 ± 0.0046	0.0064 ± 0.0041	0.0040 ± 0.0027
Thymus	0.0077 ± 0.0058	0.0116 ± 0.0059	0.0039 ± 0.0012
Thyroid	0.0060 ± 0.0039	0.0065 ± 0.0029	0.0030 ± 0.0012
Urinary bladder wall	0.0130 ± 0.0102	0.0370 ± 0.0401	0.0174 ± 0.0080
Prostate	0.0059 ± 0.0034	0.0099 ± 0.0058	0.0054 ± 0.0017
Total	0.0027 ± 0.0013	0.0036 ± 0.0015	0.0021 ± 0.0006
Effective dose equivalent (mSv/MBq)	0.0058 ± 0.0032	0.0079 ± 0.0042	0.0044 ± 0.0016
Effective dose (mSv/MBq)	0.0047 ± 0.0026	0.0066 ± 0.0036	0.0038 ± 0.0012

* Dose was significantly higher for the administration of 40 µg than for 120 µg of the peptide.

**Table 3:** Ratios of SUV_mean_ values in PCa primary tumors to surrounding normal tissue (muscle).

Dose	2 h	4 h	6 h
40 µg	4.5 ± 1.5 [2.53–6.57]	4.9 ± 0.9 [3.69–5.86]	6.4 ± 2.7 [3.63–11.67]
80 µg	5.2 ± 1.7 [3.75–7.67]	4.3 ± 1.8 [2.74–6.82]	6.5 ± 2.1 [4.45–8.65]
120 µg	6.5 ± 4.0 [3.81–12.44]	7.2 ± 3.1 [4.64–11.25]	7.0 ± 2.2 [4.73–9.11]

## Data Availability

The data are contained within the article and in the Supplementary Material.

## Supplementary Materials

The following supporting information can be downloaded at https://www.mdpi.com/article/10.3390/pharmaceutics17101323/s1: Figure S1: Anterior and posterior images of a PCa patient (Patient 8) with massive BM lesions at 2, 4, 6, and 24 h pi of [^99m^Tc]Tc-DB8 (80 µg of peptide mass). Figure S2: SPECT/CT image of the pancreas 2 h pi of [^99m^Tc]Tc-DB8 (120 µg of peptide mass) in patient No. 16. Table S1: Sequences of bombesin analogues.

## Abbreviations

The following abbreviations are used in this manuscript:

BCa	breast cancer
BMs	bone metastases
CT	computed tomography
GRPR	gastrin-releasing peptide receptor
GS	Gleason score
IHC	immunohistochemistry
LNM	lymph node metastases
MRI	magnetic resonance imaging
PCa	prostate cancer
PET	positron emission tomography
PSA	prostate-specific antigen
PSMA	prostate-specific membrane antigen
SPECT	single-photon emission computed tomography
SUV	standardized uptake value
