# Supplementary material for: Phase I Clinical Study with the GRPR-Antagonist [99mTc]Tc-DB8 for SPECT Imaging of Prostate Cancer: Does the Injected Peptide Mass Make a Difference?
Source: Pharmaceutics. 2025 Oct 12;17(10):1323. doi: 10.3390/pharmaceutics17101323 (PMC12567192; doi:10.3390/pharmaceutics17101323)
Supplement: Supplementary file 1 [file pharmaceutics-17-01323-s001.zip › pharmaceutics-3871903-supplementary.pdf]

Article

Phase I clinical study with the GRPR-antagonist [^{99m}Tc]Tc-DB8 for SPECT imaging of prostate cancer: Does the injected peptide mass make a difference?

Anna Orlova, Anastasia Rybina, Anna Medvedeva, Roman Zelchan, Olga Bragina, Liubov Tashireva, Maria Larkina, Ruslan Varvashenya, Nadejda Lushnikova, Panagiotis Kanellopoulos, Theodosia Maina, Berthold A Nock, Vladimir Tolmachev, Vladimir Chernov

Table S1. Sequences of bombesin analogues

BBN	Pyr-Gln-Arg-Tyr-Gly-Asn-Gln-Trp-Ala-Val-Gly-His-Leu-Met-NH_2
Analogues labeled with technetium-99m	
DB4	N_4-Pro-Gln-Arg-Tyr-Gly-Asn-Gln-Trp-Ala-Val-Gly-His-Leu-Nle-NH_2
DB8	N_4-AMA-DGA-DPhe-Gln-Trp-Ala-Val-Gly-His-Leu-NHEt
DB15	N_4-AMA-DGA-DPhe-Gln-Trp-Ala-Val-Sar-His-Leu-NHEt
RP527	N_3S-Gly-5-aVa-Gln-Trp-Ala-Val-Gly-His-Leu-Met-NH_2
RM26/JMV594	DPhe-Gln-Trp-Ala-Val-Gly-His-Sta-Leu-NH_2
maSSS-PEG2-RM26	mercaptoacetyl-Ser-Ser-Ser-PEG2-DPhe-Gln-Trp-Ala-Val-Gly-His-Sta-Leu-NH_2
N_4-BTG	N_4-DAsp-Pip-DPhe-Gln-Bta-Ala-Val-Gly-His-Sta-Leu-NH_2
Analogues labeled with gallium-68	
RM2	RM2, DOTA-Pip-DPhe-Gln-Trp-Ala-Val-Gly-His-Sta-Leu-NH_2
NOTA-RM26	NOTA-PEG3-DPhe-Gln-Trp-Ala-Val-Gly-His-Sta-Leu-NH_2
SB3	DOTA-AMA-DGA-DPhe-Gln-Trp-Ala-Val-Gly-His-Leu-NHEt

AMA - *p*-aminomethylaniline;

Bta - *β*-(3-benzothienyl) alanine;

DGA - diglycolic acid;

DMG - N,N-Dimethylglycine;

DOTA - 1,4,7,10-tetraazacyclododecane-1,4,7,10-tetraacetic acid;

NOTA - 1,4,7-triazacyclononane-1,4,7-triacetic acid;

N_3S - DMG-Ser-Cys;

N_4 - 6-(carboxy)-1,4,8,11-tetraazaundecane;

Pip - 4-amino-1-carboxymethyl-piperidine;

5-aVa - 5-aminovaleric acid.

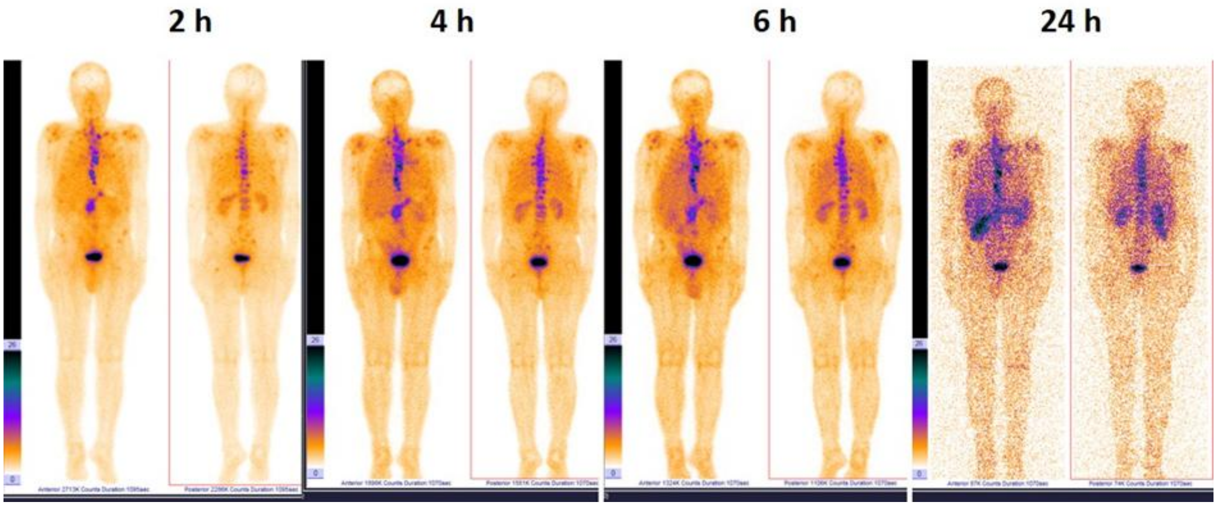

Figure S1. Anterior and posterior images of a PCa patient (Patient 8) with massive BM lesions at 2, 4, 6, and 24 h pi of [^{99m}Tc]Tc-DB8 (80 µg of peptide mass).

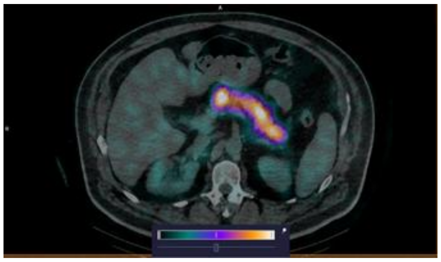

Figure S2. SPECT/CT image of the pancreas 2 hours pi of [^{99m}Tc]Tc-DB8 (120 µg of peptide mass, Patient 16).
